# An Overview of Herbal Products and Secondary Metabolites Used for Management of Type Two Diabetes

**DOI:** 10.3389/fphar.2017.00436

**Published:** 2017-07-06

**Authors:** Ajda Ota, Nataša P. Ulrih

**Affiliations:** Department of Food Science and Technology, Biotechnical Faculty, University of LjubljanaLjubljana, Slovenia

**Keywords:** type 2 diabetes mellitus, antidiabetic activity, herbal products, phenolic compounds, mechanisms of action

## Abstract

Diabetes mellitus is a common effect of uncontrolled high blood sugar and it is associated with long-term damage, dysfunction, and failure of various organs. In the adult population, the global prevalence of diabetes has nearly doubled since 1980. Without effective prevention and management programs, the continuing significant rise in diabetes will have grave consequences on the health and lifespan of the world population, and also on the world economy. Supplements can be used to correct nutritional deficiencies or to maintain an adequate intake of certain nutrients. These are often used as treatments for diabetes, sometimes because they have lower costs, or are more accessible or “natural” compared to prescribed medications. Several vitamins, minerals, botanicals, and secondary metabolites have been reported to elicit beneficial effects in hypoglycemic actions *in vivo* and *in vitro*; however, the data remain conflicting. Many pharmaceuticals commonly used today are structurally derived from natural compounds from traditional medicinal plants. Botanicals that are most frequently used to help manage blood glucose include: bitter melon (*Momordica charantia*), fenugreek (*Trigonella foenum graecum*), gurmar (*Gymnema sylvestre*), ivy gourd (*Coccinia indica*), nopal (*Opuntia* spp.), ginseng, Russian tarragon (*Artemisia dracunculus*), cinnamon (*Cinnamomum cassia*), psyllium (*Plantago ovata*), and garlic (*Allium sativum*). In majority of the herbal products and secondary metabolites used in treating diabetes, the mechanisms of action involve regulation of insulin signaling pathways, translocation of GLUT-4 receptor and/or activation the PPARγ. Several flavonoids inhibit glucose absorption by inhibiting intestinal α-amylase and α-glucosidase. In-depth studies to validate the efficacies and safeties of extracts of these traditional medicinal plants are needed, and large, well designed, clinical studies need to be carried out before the use of such preparations can be recommended for treatment and/or prevention of diabetes. The main focus of this review is to describe what we know to date of the active compounds in these, along with their glucose-lowering mechanisms, which are either through insulin-mimicking activity or enhanced glucose uptake.

## Introduction

### Diabetes: Definition and Description

Diabetes mellitus is a group of metabolic diseases that are characterized by hyperglycemia and arise from defects in insulin secretion, insulin action, or both. Chronic hyperglycemia, or raised blood sugar, is a common effect of uncontrolled diabetes, and this is associated with long-term damage, dysfunction, and failure of various organs, especially the eyes, kidneys, nerves, heart, and blood vessels (WHO expert consultation, 2002).

There are two main types of diabetes, which are characterized by progressive β-cell death. Type 1 diabetes was previously known as insulin-dependent, juvenile or childhood-onset diabetes, and it is characterized by deficient insulin production that requires daily administration of insulin. This results from cellular-mediated autoimmune destruction of the β-cells of the pancreas. Type 2 diabetes was previously known as non-insulin-dependent or adult-onset diabetes, and it results from the ineffective use of insulin by the body. Several risk factors are known to be involved in the development of type 2 diabetes, including: genetic factors (i.e., family history), obesity, poor diet, insufficient physical activity, advancing age, ethnicity, high blood glucose during pregnancy, hypertension, and dyslipidemia (Newman et al., 1987; Kaprio et al., 1992; Matsuda and Kuzuya, 1994; Chehade et al., 2013).

Gestational diabetes represents a third class of diabetes, and this arises from glucose intolerance, with an onset during pregnancy. Women with gestational diabetes are at an increased risk of complications during pregnancy and at delivery. Although gestational diabetes is a temporary condition, it carries a long-term risk of type 2 diabetes (Bellamy et al., 2009).

According to the World Health Organization, an estimated 422 million adults were living with diabetes in 2014. In the adult population, the global prevalence of diabetes has nearly doubled since 1980. Over the past decade, diabetes prevalence has risen more rapidly in low-income and middle-income countries than in high-income countries. Diabetes caused 1.5 million deaths in 2012, with hyperglycemia causing an additional 2.2 million deaths, as it is associated with increased risk of cardiovascular and other diseases. Although separate global estimates of diabetes prevalence for type 1 and type 2 diabetes are not available, the majority of people with diabetes are affected by type 2 diabetes. Also of concern is that previously type 2 diabetes was diagnosed almost entirely among adults, while now it also occurs in children (WHO, 2016). Without effective prevention and management programs, further significant rises in diabetes will have grave consequences on the health and lifespan of the world population (International Diabetes Federation, 2013).

Oxidative stress is an acknowledged pathogenic mechanism in the development and progression of diabetes and its complications, which can arise as a result of increased free radical production and impaired antioxidant defenses (Ceriello, 2000; Maritim et al., 2003; King and Loeken, 2004; Vos et al., 2012). There are several known mechanisms whereby hyperglycemia contributes to the pathogenesis of diabetic complications: non-enzymatic glycosylation of proteins and lipids; protein kinase C activation; activation of the polyol pathway; and auto-oxidation of glucose (Baynes and Thorpe, 1999; Ceriello, 2000; Maritim et al., 2003; Aronson, 2008).

Oxidative stress and other stresses caused by overnutrition (endoplasmic reticulum stress, lipotoxicity, and glucotoxicity) are thought to induce inflammatory response that plays an important role in the pathogenesis of type 2 diabetes (Shoelson et al., 2006; Donath and Shoelson, 2011; Ye and Driver, 2016). Recent studies have also shown the pathological involvement of the immune system in type 2 diabetes, that is, based on the presence of autoantibodies against β cells, self-reactive T cells and evidence of beneficial effect of some anti-inflammatory and immunomodulatory therapy, being viewed as an autoinflammatory disease (Itariu and Stulnig, 2014). Nutritional interventions with food supplements as a form of complementary therapy in patients with diabetes are being used increasingly to counter these effects (Shane-McWhorter, 2005; Campbell, 2010; Ho et al., 2013; Facchinetti et al., 2014). In this review, botanicals that are most frequently promoted to help manage blood glucose levels and can be found marketed as food supplements promoting antidiabetic activity were selected and their mechanism of action described.

### Food Supplements in Diabetes

Food supplements are concentrated sources of nutrients or other substances that can have nutritional or physiological effects, where the purpose is to supplement the normal diet. Food supplements are marketed in “dose” forms, such as pills, tablets, capsules, and liquids in measured doses. Supplements can be used to correct nutritional deficiencies or to maintain adequate intake of certain nutrients. However, in some cases excessive intake of vitamins and minerals might be harmful or cause unwanted side effects; therefore, indications for their maximum levels are necessary to ensure their safe use in food supplements (EFSA, 2015b). Recently, antioxidants have been extensively used to counter or overcome the effects of excess reactive oxygen species, which are involved in several pathologies, including diabetes and obesity. Another therapeutic approach for treating type 2 diabetes is the use of phytochemicals to lower the glucose absorption by inhibiting intestinal carbohydrate-hydrolysing enzymes α-amylase and α-glucosidase (Sales et al., 2012; Shori, 2015). Several phytochemicals were also been reported to reduce inflammatory compounds involved in the pathology of type 2 diabetes.

## Herbal Products and Secondary Metabolites

### Herbal Products with Hyperglycemic Actions

Botanicals have been used for medicinal purposes through much of human history (Petrovska, 2012). Botanicals and their derived preparations made from plants, algae, fungi, and lichens have become widely available on the EU market in the form of food supplements (EFSA, 2015a).

Many pharmaceuticals commonly used today are structurally derived from the natural compounds that are found in traditional medicinal plants. For example, the development of the antihyperglycemic drug metformin can be traced to the traditional use of *Galega officinalis* to treat diabetes (Evans and Bahng, 2014). Botanicals that are most frequently promoted to help manage blood glucose levels include bitter melon (*Momordica charantia*), fenugreek (*Trigonella foenum graecum*), gurmar (*Gymnema sylvestre*), ivy gourd (*Coccinia grandis*), nopal (*Opuntia* spp.), ginseng, Russian tarragon (*Artemisia dracunculus*), cinnamon (*Cinnamomum cassia*), psyllium (*Plantago ovata*), and garlic (*Allium sativum*) (Cefalu et al., 2008).

#### Bitter Melon (*M. charantia* L.)

*Momordica charantia* is a climbing plant that belongs to the family Cucurbitaceae and is commonly known as bitter gourd or bitter melon. All parts of the plant have a bitter taste, including the fruit, which have a warty texture that resembles a small cucumber. *M. charantia* is cultivated throughout the tropics and subtropics (Grover and Yadav, 2004). The main phytochemical constituents of bitter melon that have reported hypoglycemic actions are the cucurbitane-type triterpenoids charantin (a steroidal glycoside that is an equal mixture of stigmasterol glucoside and β-sitosterol glucoside), karaviloside IX, momordicoside S and its aglycones momordicosides A, B, Q, R, and T, and also polypeptide-p, vicine, and the ribosome-inactivating protein momordin (Tan et al., 2008; Joseph and Jini, 2013). Several mechanisms of action have been proposed for the hypoglycemic actions of bitter melon extracts. Studies have shown that these can inhibit intestinal absorption of glucose (Grover and Yadav, 2004; Chaturvedi, 2012), suppress key glucogenic enzymes (Shibib et al., 1993), and decrease hepatic gluconeogenesis (Tsai et al., 2012). It has been proposed that *M. charantia* enhances the activity of the AMP-activated protein kinase (AMPK) pathway (which is an important cellular regulator of lipid and glucose metabolism), and reduces expression of phosphoenolpyruvate carboxykinase (PEPCK; which results in reduced glucose levels; Shih et al., 2014). Polypeptide-p is sometimes referred as “plant insulin,” and it is one of the few of these active compounds that have been studied in clinical trials. Polypeptide-p consists of 166 amino-acid residues and closely resembles bovine insulin (Khanna et al., 1981; Efird et al., 2014). Polypeptide-p is a very effective hypoglycemic agent when administered subcutaneously (Joseph and Jini, 2013). Clinical study investigating the effect of subcutaneous injection of polypeptide-p resulted in a statistically significant drop in mean blood sugar levels (Baldwa et al., 1977). Recent study demonstrated that administration of a cucurbitane-type triterpenoid isolated from *M. charantia* named compound K16 reduced blood glucose and blood lipids in murine model, while improving glucose tolerance. Compound K16 also upregulated the expression of several of insulin signaling pathway-associated proteins (Jiang et al., 2016). Only a few randomized controlled trials of bitter melon have been conducted. According to Efird et al. (2014) out of 21 reviewed studies less than 20% received a quality score of two or greater on the Jadad scale. Although evidence suggests possible beneficial effects of extracts of bitter melon and its active compounds in the prevention and control of diabetes, future clinical studies are needed to confirm this.

#### Fenugreek (*T. foenum graecum* L.)

**FIGURE 1:**
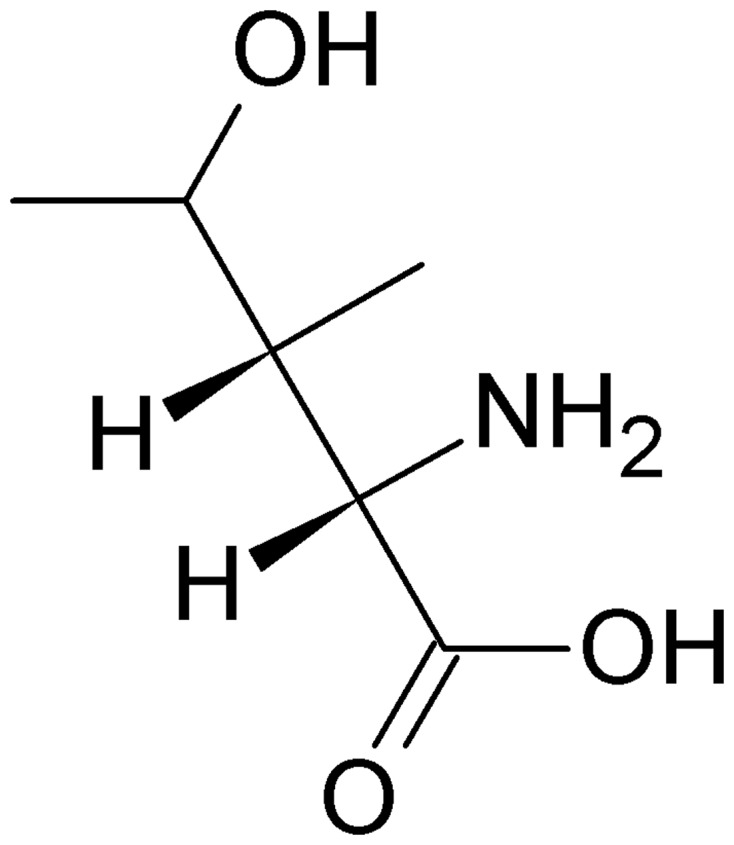
Structure of 4-hydroxyisoleucine, a branched-chain amino-acid derivative that is only found in plants.

Fenugreek is a plant of the Fabaceae family that is native to India, China, and North Africa (Prabhakar and Doble, 2011). The most studied bioactive compounds from fenugreek with reported hypoglycemic actions are diosgenin (3b-hydroxy-5-spirostene), 4-hydroxyisoleucine (**Figure 1**), and the soluble dietary fiber fraction of fenugreek seeds (Fuller and Stephens, 2015).

Diosgenin in fenugreek is a major aglycone of saponin, and the reported hypoglycemic mechanisms of its action include renewal of pancreatic β-cells and stimulation of insulin secretion (Kalailingam et al., 2014), antioxidative effects (Son et al., 2007), and promotion of adipocyte differentiation and enhancement of insulin-dependent glucose uptake (Uemura et al., 2010). 4-Hydroxyisoleucine is a branched-chain amino-acid derivative that is only found in plants, and it represents the majority of the total content of free amino acids in fenugreek seeds (Fuller and Stephens, 2015). It has been shown that the insulinotropic and antidiabetic properties of 4-hydroxyisoleucine act through stimulation of glucose-dependent insulin secretion and reduction of insulin resistance in muscle and/or liver (Jetté et al., 2009). Fenugreek seeds are also a rich source of fiber (50–65 g fiber/100 g seeds). The soluble dietary fiber fraction of fenugreek (i.e., galactomannan) has been shown to enhance glycemic control. This effect has been attributed to inhibition of lipid-hydrolyzing and carbohydrate-hydrolyzing enzymes in the digestive system (Hannan et al., 2007). Galactomannan also reduces the rate of glucose uptake through its actions as a significant physical barrier to glucose diffusion (Srichamroen et al., 2009). Clinical studies reported that intake of fenugreek seeds significantly changed fasting blood glucose, 2 h postload glucose and hemoglobin A1c (HbA1c; Neelakantan et al., 2014). Results of a recent study conducted on men and women with prediabetes strongly suggests that the enhancement of insulin levels is due to insulinotropic effect of fenugreek and suggest that the mode of action is a result of alkaloids present (Gaddam et al., 2015). Although results from clinical trials support beneficial effects of fenugreek seeds on glycemic control in persons with diabetes, trials with better methodology quality and well characterized preparation of sufficient dose are needed to provide more conclusive evidence. With its hypoglycemic and antidyslipidemic effects, fenugreek represents an attractive new candidate for treatment of type 2 diabetes, obesity, and dyslipidemia, the key components of metabolic syndrome.

#### Gurmar [*G. sylvestre* (Retz.) R.Br. ex Sm.]

*Gymnema sylvestre* is a large woody climbing plant that grows in the dry forests of India. In Hindu folklore, chewing gymnema leaves results in the inability to taste sweetness. It is therefore also known as “gurmar,” which means “destroyer of sugar” in Hindi (Yeh et al., 2003). The active compounds that can be extracted from this plant are the gymnemic acids, of which there are several (gymnemic acids I–VII, gymnemosides A–F; Tiwari et al., 2014) and the gymnemasaponins (Leach, 2007; Khramov et al., 2008). Another compound with sugar suppression activity that has been isolated from *G. sylvestre* is gurmarin, a 35-amino-acid peptide (Imoto et al., 1991). The atomic arrangement of gymnemic acids on the taste buds are similar to that of sugar molecules, and therefore their binding to the receptors on the taste buds prevents receptor activation by the sugar molecules in the food, which prevents the taste of sweetness.

The proposed mechanisms for the hypoglycemic action of gymnemic acids might be increased secretion of insulin from the pancreas and promotion of islet cell regeneration (Baskaran et al., 1990). Gymnemic acids can also delay glucose absorption in the blood and prevent absorption of sugar molecules by the intestine, which leads to reductions in blood sugar levels (Tiwari et al., 2014). Although several studies have reported antidiabetic effects and sugar inactivation properties of gurmar (Kumar et al., 2014; Li et al., 2015; Kamble et al., 2016), and recent interventional, randomized, double blind clinical study (NCT02370121) reported statistically significant reduction in body weight, reduced BMI and lower values for VLDV (ClinicalTrials.gov, 2016), clinical approval and scientific validation remain necessary before its use can be approved in the treatment of patients with diabetes.

#### Ivy gourd [*C. grandis* (L.) Voigt, syn. *Coccinia indica* Wight & Arn.]

Ivy gourd (*C. grandis*) is a perennial creeper that belongs to the Cucurbitaceae family. It has been traditionally used in Ayurvedic practice as an antidiabetic drug. The proposed mechanism of action of *C. grandis* is not well understood, although it appears to be an insulin mimetic (Kamble et al., 1998). Extracts of *C. grandis* are believed to have hypoglycemic effects through an insulin-secreting effect or through an influence on the enzymes involved in glucose metabolism (Patel et al., 2012). Despite the broad use of *C. grandis* in traditional medicine, very few systematic clinical studies have been reported to date that have examined its therapeutic properties. Human trials have shown that the active components of a *C. grandis* extract can reduce elevated levels of the enzymes glucose-6-phosphatase and lactase dehydrogenase in the glycolytic pathway, and restore lipoprotein lipase activity in the lipolytic pathway, with the control of hyperglycemia in diabetes (Hossain et al., 1992; Kamble et al., 1998; Kuriyan et al., 2008). Double-blind clinical trial conducted on 61 healthy individuals showed statistically significant difference in postprandial blood sugar levels after 1 and 2 h confirming a blood sugar lowering effect of *C. grandis* (Munasinghe et al., 2011). The data from animal and human trials are promising, and the reported hypoglycemic action of *C. grandis* might indicate its use as a dietary adjunct in the treatment of patients with diabetes.

#### Nopal (*Opuntia* spp.)

The genus *Opuntia* is commonly known as prickly pear cactus, and this includes species that produce the nutritious fruit and young, edible cladodes (stem pads) that have been used for both nourishment and medicine for hundreds of years (González-Stuart, 2013; Cota-Sánchez, 2016). The prickly pear cactus is traditionally used to treat diabetes in the form of a blended “shake” that is prepared from young cladodes (Becerra-Jiménez and Andrade-Cetto, 2012). Nopal is rich in highly soluble fiber and pectin, which can affect intestinal glucose uptake, thus partly explaining its hypoglycemic actions (Frati et al., 1990; Evans and Bahng, 2014). Several studies have confirmed that a total extract and a juice from the plant can have antihyperglycemic effects (Ibañez-Camacho et al., 1983; Becerra-Jiménez and Andrade-Cetto, 2012). Data from animal studies using extracts without fiber and pectin that have shown antihyperglycemic actions have suggested additional modes of action (Trejo-González et al., 1996; Andrade-Cetto and Wiedenfeld, 2011). Polysaccharide ODP-Ia has been isolated from *Opuntia dillenii* Haw., and this was shown to have antihyperglycemic effects through the protection of the liver from peroxidation damage and through the maintenance of tissue function, thereby improving the sensitivity of target cells to insulin (Zhao et al., 2011). Recent study conducted on murine model suggested that *Opuntia ficus-indica* treatment acts by inhibiting glucose absorption from the intestine and enhancing glucose uptake from insulin-sensitive muscle cells through the AMPK/p38 MAPK signaling pathway (Leem et al., 2016). Findings of a recent study conducted on Mexican patients with type 2 diabetes suggest that *O. ficus-indica* (L.) Mill. could reduce postprandial blood glucose and serum insulin, as well as increase antioxidant activity in healthy people and patients with type 2 diabetes (López-Romero et al., 2014). Although preliminary human and animal trials have suggested that certain *Opuntia* species have hypoglycemic properties, more clinical studies are necessary to determine the true benefits of these plants as botanical interventions in the treatment of patients with type 2 diabetes.

#### Ginseng: Korean or Asian ginseng (*Panax ginseng* C.A. Mey.) and American ginseng (*Panax quinquefolius* L.)

Out of 14 identified ginseng species to date, Korean red ginseng (*P. ginseng*) and American ginseng (*P. quinquefolius*) have been the most extensively used and studied (Christensen, 2009). The part of the plant that is mainly used for medicinal purposes is the roots, although other parts are also being investigated for antidiabetic effects (Xie et al., 2002; Dey et al., 2003). The active components in ginseng include polysaccharides and polyacetylenes, although the majority of its pharmacological activity has been attributed to the ginsenosides, a group of triterpenoid saponins (Christensen, 2009; Wee et al., 2011). Indeed, more than 150 naturally occurring ginsenosides have been isolated from the roots, leaves, stems, fruit, and flower heads of various *Panax* species (Christensen, 2009). Several clinical trials and animal studies have demonstrated that ginseng and ginsenosides can lower blood glucose, to increase insulin sensitivity and regulate lipid metabolism (Cho et al., 2006; Vuksan et al., 2008). The proposed mechanism of the modulation of metabolic processes by ginsenosides is their activation of the peroxisome proliferator-activated receptors (PPARs) that regulate glucose and lipid metabolism, and the transcription of proteins involved in glucose and fatty-acid uptake (Auwerx et al., 1996). Recent studies have shown that ginsenosides activate AMPK pathway, resulting in suppression of hepatic gluconeogenesis and steatosis (Gui et al., 2016). Additional potential health effects of ginsenosides include anticarcinogenic, immunomodulatory, anti-inflammatory, anti-allergic, anti-atherosclerotic, antihypertensive, and antidiabetic effects, as well as effects on the central nervous system (Christensen, 2009). Meta-analysis of 16 randomized controlled trials in people with and without diabetes concluded that ginseng modestly yet significantly improved fasting blood glucose in people with and without diabetes (Shishtar et al., 2014). For better assessment of ginseng’s anti-diabetic efficacy, larger and longer randomized controlled clinical studies, using standardized ginseng preparations are warranted.

#### Russian tarragon (*A. dracunculus* L.)

*Artemisia dracunculus* L., or Russian tarragon, is a perennial herb from the Asteraceae (daisy) family. French tarragon (which is also known as German tarragon) and Russian tarragon are the two main reported cultivars for this species (Obolskiy et al., 2011). *A. dracunculus* has been widely used in traditional medicine because of its potential antimicrobial and antioxidant activities (Lopes-Lutz et al., 2008; Ahameethunisa and Hopper, 2010). The most important groups of biologically active secondary metabolites in *A. dracunculus* essential oils are coumarins, flavonoids, and phenolic acids (Obolskiy et al., 2011). Several studies have reported antidiabetic effects of *A. dracunculus* (Govorko et al., 2007; Kheterpal et al., 2014; Ribnicky et al., 2014; Aggarwal et al., 2015). A study by Aggarwal et al. (2015) demonstrated that a well-characterized extract of *A. dracunculus* L. known as PMI-5011 can trigger insulin release from primary β-cells, as well as protect β-cells, which contributes to the preservation of metabolic homeostasis of insulin and β-cells. It has also been shown that PMI-5011 promotes decreased glucose and insulin levels in animal models and improves insulin signaling in primary human skeletal muscle cells (Kheterpal et al., 2014; Obanda et al., 2014; Vandanmagsar et al., 2014). PMI-5011 treatment also led to an inhibition of cytokine-induced activation of inflammatory signaling pathways (Vandanmagsar et al., 2014). Anti-inflammatory effect of ethanol extract was also demonstrated in murine model (Eidi et al., 2016). These data indicate that this PMI-5011 *A. dracunculus* L. extract represents a promising botanical treatment for patients with diabetes. The most recent randomized, double blind, placebo-controlled clinical trial (NCT02330341) evaluating the effect of *A. dracunculus* on glycemic control, insulin sensitivity, and insulin secretion in patients with impaired glucose tolerance reported significantly decreased systolic blood pressure, glycated HbA1c, area under the curve of insulin, and total insulin secretion with a significant increase in HDL-C levels (Méndez-del Villar et al., 2016).

#### Cinnamon (*Cinnamomum* spp.)

*Cinnamomum* (cinnamon) is a genus of the Lauraceae family. To date, about 250 species of cinnamon have been identified (Medagama, 2015). Several preclinical and clinical investigations have demonstrated that extracts of cinnamon species can have antidiabetic activities, and have investigated their mechanism of action (Akilen et al., 2012; Chen et al., 2012; Cheng et al., 2012; Verspohl et al., 2005). The procyanidin oligomers are thought to be responsible for the antidiabetic activity of cinnamon (Lu et al., 2011; Chen et al., 2012). The mechanism behind the antidiabetic actions of cinnamon species is not fully understood yet. Different hypoglycemic effects have been reported for different cinnamon species. Chen et al. (2012) reported that a *C. cassia* extract can promote lipid accumulation in adipose tissue and liver, whereas a *Cinnamomum tamala* (Buch.-Ham.) T. Nees & C.H. Eberm. extract mainly improved the insulin concentrations in the blood and pancreas. They defined the different antidiabetic effects according to the diverse procyanidin oligomer components in these extracts (Chen et al., 2012). Improved insulin resistance and lipid metabolism have been reported for a water extract of cinnamon, which was believed to be through activation of PPARs (Sheng et al., 2008). Decreased gene expression of two major regulators of hepatic gluconeogenesis has been reported for a cinnamon water extract (i.e., PEPCK and glucose-6-phosphatase), as also reported for a murine model (Cheng et al., 2012). Cinnamon also contains anti-inflammatory compounds that reduce production of prostaglandin-E2, interleukin 6 and nitric oxide (NO). *Trans*-cinnamaldehyde exhibited the strongest activity on NO production (Tung et al., 2008). A study on rat model of gestational diabetes showed hypoglycemic action of cinnamaldehyde by increasing insulin secretion and sensitivity through activating the antioxidant defense system, suppressing pro-inflammatory cytokines production and upregulating PPARγ gene expression (Hosni et al., 2017). Review of clinical trials, evaluating the experimental evidence available for cinnamon in improving glycemic targets concluded that cinnamon has the potential to be a useful add-on therapy in the managing type 2 diabetes, but further trials are needed to establish its efficacy and safety (Medagama, 2015). Data from different animal and human studies warrant continued investigations into the benefits of different cinnamon extracts supplementation on the prevention and treatment of type 2 diabetes and gestational diabetes.

#### Garlic (*A. sativum* L.)

**FIGURE 2:**
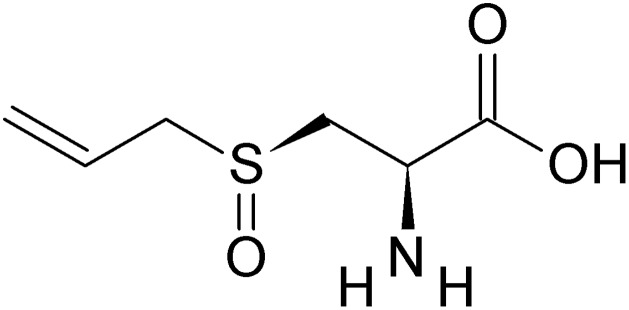
Structure of alliin.

Garlic (*A. sativum*) is a member of the Amaryllidaceae family, along with onions, chives, and shallots (Iciek et al., 2009). In addition to promoting total antioxidant levels and catalase activity, the antidiabetic potential of garlic includes: hyperinsulinemia, hypoglycemia, hypocholesterolemia, hypotriglyceridemia, and anti-glycation and anti-lipid-peroxidation actions (Thomson et al., 2016). The active ingredients of garlic that have been attributed to its beneficial effects are mainly volatile sulfur compounds, like alliin (**Figure 2**), allicin, diallyl disulfide, diallyl trisulfide, diallyl sulfide, *S*-allyl cysteine, ajoene, and allyl mercaptan (Padiya and Banerjee, 2013; Bayan et al., 2014).

Both fresh and aged garlic and its bioactive compounds have been extensively studied for their antihyperglycemic actions both in experimentally induced and genetic animal models of diabetes (Padiya et al., 2011; Shiju et al., 2013; Al-Qattan et al., 2016; Sathibabu Uddandrao et al., 2016; Thomson et al., 2016) and in human studies (Ashraf et al., 2011; Atkin et al., 2016). Garlic has been shown to improve insulin sensitivity and the associated metabolic syndrome in animal models (Padiya et al., 2011). Clinical trial studying the effect of oral administration of raw garlic on type 2 diabetic patients showed a significant reduction in blood glucose level, lipid metabolism and significant improvement in superoxide dismutase, catalase, and glutathione peroxidase in erythrocytes of diabetic patients (Mirunalini et al., 2011). Several studies have also reported increased insulin secretion upon administration of garlic or garlic extracts/preparations (Eidi et al., 2006; Liu et al., 2006). Islam and Choi (2008) speculated that the higher insulin production is a result of the actions of allixin, *S*-allyl cysteine sulfoxide, and diallyl trisulfide. Recent studies of *S*-allyl cysteine, the main organosulfur bioactive molecule in aged garlic extract, demonstrated its anti-diabetic, antioxidant, anti-inflammatory and neuroprotective properties (Baluchnejadmojarad et al., 2017; Zarezadeh et al., 2017). Studies in animal models and preliminary human studies have indicated beneficial effect of garlic and garlic extracts in the treatment of patients with diabetes and related metabolic disorders; however, more clinical trials are warranted to further explore the benefits of garlic for patients with type 2 diabetes.

#### Psyllium (*P. ovata* Forssk.)

*Plantago* spp. belongs to the Plantaginaceae family, which comprises approximately 275 annual and perennial species that are distributed throughout the world. Some species are particularly valuable in the nutraceutical and pharmaceutical industries due to the mucilaginous product that is derived from the seed husk, which is known as psyllium (Gonçalves and Romano, 2016). The most abundant polysaccharide in psyllium are the heteroxylans (Thakur and Thakur, 2014). Preparations obtained from seed husks of *P. ovata* are an excellent source of soluble fiber (Moreno et al., 2003). As psyllium is less readily fermented, it causes fewer abdominal problems and it is better tolerated than other fiber supplements (Pal and Radavelli-Bagatini, 2012). The proposed glucose-lowering mechanisms of psyllium are: slowed access of glucose to the small intestine; delayed gastric emptying; and actions on carbohydrate digestion and absorption (Pastors et al., 1991; Anderson et al., 1999). Clinical study evaluating the effects of psyllium in type 2 diabetic patients reported significant decrease of glucose absorption and reduction of total and LDL cholesterol in the presence of psyllium, indicating its beneficial therapeutic effect in the metabolic control of type 2 diabetics (Sierra et al., 2002).

#### Ginger (*Zingiber officinale* Roscoe)

Ginger belongs to Zingiberaceae family and its rhizome is widely used as a spice all over the world. It has been traditionally used as herbal medicine to treat cancer, rheumatism, toothache, digestive health, and diabetes (Park and Pezzuto, 2002; Ali et al., 2008). Its components gingerols, shogaols, paradols, and zingiberene exhibit antioxidative effect, glucose and lipid lowering effects, as well as immunomodulatory, anti-inflammatory, antiapoptosis effect (Jolad et al., 2004; Ali et al., 2008; Arablou et al., 2014). The anti-inflammatory action of ginger is attributed to gingerols, shogaols, and diarylheptanoids that are thought to inhibit the activity of cyclooxygenase, inducible NO synthase and lipoxygenase suppress prostaglandin synthesis and interfere in cytokine signaling. Recent study reported improved insulin sensitivity and reduced total cholesterol and triglycerides as well as reduced C-reactive protein and prostaglandin E2 in patients with type 2 diabetes (Arablou et al., 2014). A double-blind, placebo-controlled, randomized clinical trial conducted on patients with type 2 diabetes who did not receive insulin showed that ginger supplementation significantly reduced serum triglyceride and reported a minor beneficial effect on serum glucose (Shidfar et al., 2015).

Different studies revealed more than 100 plant species generally used for treatment of diabetes (Chauhan et al., 2010; Eddouks et al., 2014; Mamun-or-Rashid et al., 2014). The majority of the experiments confirmed their beneficial effect in the management of diabetes mellitus. Among the plants used for the diabetes, the most commonly studied species are described above, while other common plants used to treat diabetes that are available everywhere and their active phytochemicals are reported in **Table 1**.

**Table 1:** List of most common plants and their active phytochemicals having antidiabetic activity.

Medicinal plant	Active phytochemical(s)	Reference
*Aegle marmelos*	Aegelin, coumarins, alkaloids	Chauhan et al., 2010
*Allium cepa*	Allyl sulfide	Chauhan et al., 2010
*Aloe vera*	Aloin, aloe-emodin, pseudoprototinosaponin AIII, prototinosaponin AIII	Patel et al., 2012; Eddouks et al., 2014
*Arctium lappa*	Sitosterol-beta-D-glucopyranoside	Chan et al., 2011
*Cannabis sativa*	Cannabinoids, cannabinol	Chauhan et al., 2010; Di Marzo et al., 2011
*Lycium barbarum*	Polysaccharide	Zhao et al., 2005
*Morus alba*	Moran A	Chauhan et al., 2010
*Olea europaea*	Triterpenoids	Eddouks et al., 2014
*Oryza sativa*	Glycan	Chauhan et al., 2010
*Psidium guajava*	Vescalagin, strictinin, isostrictinin, pedunculagin	Patel et al., 2012; Eddouks et al., 2014
*Punica granatum*	Gallic acid, ellagic acid	Farzaei et al., 2017
*Stevia rebaudiana*	Stevioside	Patel et al., 2012
*Ziziphus spina-christi*	Christinin-A	Patel et al., 2012

### Secondary Metabolites

#### Plant Polyphenols

Polyphenols are a large and heterogeneous group of phytochemicals that can be found in plant-based foods, such as tea, coffee, wine, cocoa, cereal grains, legumes, fruit, and berries (Hanhineva et al., 2010). Polyphenols are divided into flavonoids, phenolic acids, stilbenes, and lignans. Flavonoids can be further divided into flavones, flavonols, flavanols, flavanones, isoflavones, and anthocyanins (Manach et al., 2004). Evidence from numerous animal studies support antidiabetic properties for some of the dietary polyphenols, which suggests their potential for prevention and management of type 2 diabetes (Kim et al., 2016). Several mechanisms might contribute to their hypoglycemic effects, including: inhibition of carbohydrate digestion and glucose absorption in the intestine; stimulation of insulin secretion from pancreatic β-cells; modulation of glucose release from the liver; activation of insulin receptors and glucose uptake in insulin-sensitive tissues; and modulation of intracellular signaling pathways and gene expression (Hanhineva et al., 2010; Bahadoran et al., 2013).

##### Resveratrol

Resveratrol (3,5,4′-trihydroxy-trans-stilbene) is a phytoalexin that is naturally synthesized by plants as a defense mechanism that is triggered in response to infection and injury by fungi, bacteria, UV irradiation, and other plant stresses (Park and Pezzuto, 2015). Resveratrol is found in berries, grape skins, red wine, Japanese knotweed, peanuts, and roots of rhubarb (Kim et al., 2016), and it has two isomeric forms (Ali et al., 2010). Several studies have reported blood-glucose-lowering effects of resveratrol in animal models (Jiang et al., 2013; Szkudelski and Szkudelska, 2015). Several mechanisms of the antidiabetic action of resveratrol have been reported: improvement of insulin sensitivity; enhancement of GLUT4 translocation; reduction in oxidative stress, regulation of carbohydrate-metabolizing enzymes; activation of sirtuin 1 (SIRT1) and AMPK; and reduction in the expression of adipogenic genes (Bagul and Banerjee, 2015; Bitterman and Chung, 2015). The deacetylase activity of SIRT1 is believed to deacetylate and suppress Foxo1-induced transactivation of pyruvate dehydrogenase lipoamide kinase 4 (PDK4), a negative regulator of the glycolytic enzyme pyruvate dehydrogenase (Sin et al., 2015).

Although they have been limited to date, clinical studies combined with data from *in vitro* and animal studies have indicated the potential antidiabetic effects of resveratrol, as well as its effects on related metabolic disorders, although further studies are warranted specifically regarding its optimal doses (Wang et al., 2014).

##### Flavonoids

**FIGURE 3:**
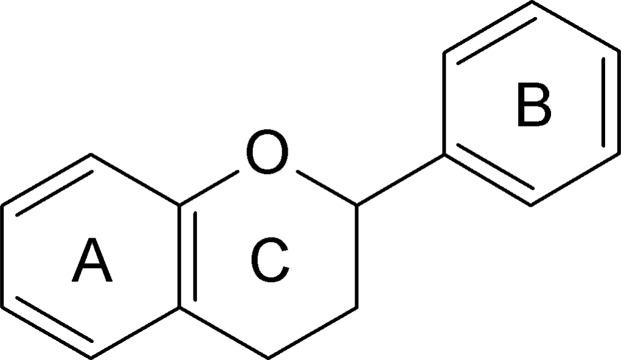
General structure of the 15-carbon skeleton of flavonoids, which consists of two phenyl rings (A, B) and a heterocyclic ring (C).

Flavonoids are secondary metabolites of plants and fungi that have a 15-carbon skeleton (**Figure 3**) that contains two phenyl rings and a heterocyclic ring, and they are primarily known as the pigments responsible for producing the many colors of flowers, fruit, and leaves (Kawser Hossain et al., 2016). Several clinical and experimental studies have suggested that flavonoids have positive effects in the treatment, prevention, and alleviation of various diseases (Havsteen, 2002; Jayaprakasam et al., 2006; Lee et al., 2007). The proposed mechanisms of action of flavonoids include antioxidant actions, central nervous system effects, alterations to gut transport, fatty-acid sequestration and processing, PPAR activation, and increased insulin sensitivity (Prasain et al., 2010).

**FIGURE 4:**
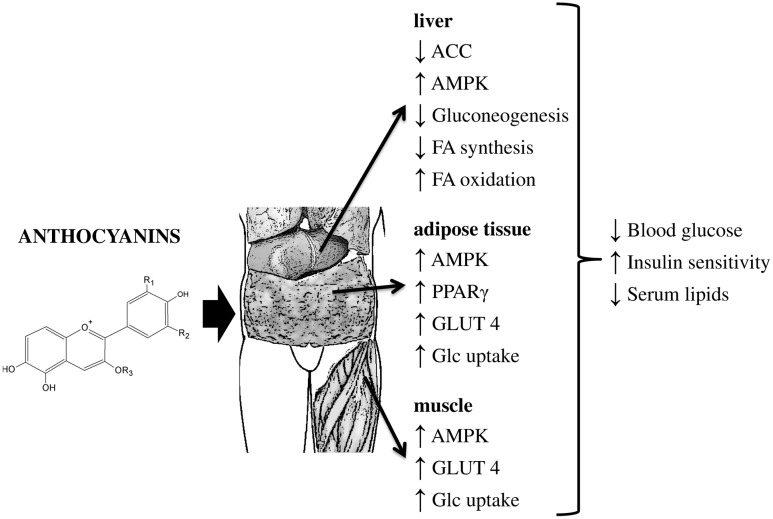
Proposed biological mechanisms underlying the actions of anthocyanins on hyperglycemia and insulin sensitivity. ACC, acetyl-CoA carboxylase; FA, fatty acid; Glc, glucose; GLUT4, glucose transporter 4; PEPCK, phosphoenolpyruvate carboxykinase.

The anthocyanins are flavonoids that are of great nutritional interest as they are one of the major classes of the widely consumed dietary polyphenols in fruit and vegetables (Guo and Ling, 2015). They are especially abundant in dark-colored fruit, such as berries (Pojer et al., 2013; Tsuda, 2016). Over the years, the antidiabetic potential of anthocyanin-rich foods has been well documented (Guo and Ling, 2015). A randomized, double-blinded, placebo-controlled clinical study reported by Stull et al. (2010) showed that a daily dietary supplement with bioactives from whole blueberries promoted improved insulin sensitivity in obese, non-diabetic, and insulin-resistant participants. Several studies have been conducted to investigate the impact of the consumption of purified anthocyanins on the development and progression of type 2 diabetes mellitus (**Figure 4**). These have shown improvements to whole-body insulin sensitivity and significant reduction of fasting blood glucose (Stull et al., 2010), as well as increased serum adiponectin concentrations (Liu et al., 2014). It has been proposed that cyanidin 3-glucoside and its metabolite protocatechuic acid have insulin-like activities through regulation of internalization of glucose via the PPARγ signaling pathway. The increased PPARγ activity caused by cyanidin 3-glucoside resulted in upregulation of *Glut4* and translocation of GLUT4 to the membrane, as well as enhanced adiponectin secretion in human omental adipocytes (Scazzocchio et al., 2011). Numerous studies have suggested that anthocyanins target AMPK in skeletal muscle and liver, which results in increased glucose uptake and inhibition of gluconeogenesis (Kurimoto et al., 2013). Simultaneously, acetyl-CoA carboxylase is inactivated and PPARα, acyl-CoA oxidase, and carnitine palmitoyltransferase-1A are upregulated in the liver, which leads to a reduction of the lipid content and enhanced insulin sensitivity (Tsuda, 2016).

One of the limitations of anthocyanins compared to the other flavonoids is their relatively low bioavailability that varies depending on the food matrix, and on other antioxidants and macronutrients in the foods consumed, which consequently affect the absorption and antioxidant potential of anthocyanins (Yang et al., 2011). Celli et al. (2016) proposed a strategy to overcome the limitations in anthocyanin delivery, using gastro-retentive systems that can promote sustained release of anthocyanins. The different structures and the heterogeneity of anthocyanins in berries and their health-promoting effects in synergy with other compounds warrant further studies to fully elucidate and understand the effects of anthocyanins in diabetes and on health in general (Pojer et al., 2013). The majority of epidemiological studies have associated dietary polyphenols with lower risk of type 2 diabetes mellitus; however, these data have been inconsistent. Further well-designed studies are necessary before any specific health claims can be made.

Kaempferol, a dietary flavonol abundant in *Ginkgo biloba* L., grapefruit, broccoli, kale, tea, and many other edible plants. It was found to have anti-oxidant and anti-inflammatory effects in various disease models, including those for diabetes (Calderón-Montaño et al., 2011). Several mechanisms of its anti-diabetic action were reported. Kaempferol inhibits NF-κB pathway activation, thus inhibits hepatic inflammation, which is contributing to the improvement of insulin signaling defect in diabetes (Luo et al., 2015). Kaempferol was found to have protective effect on pancreatic β-cell. Its protective action is associated with improved cAMP signaling, inhibited cellular apoptosis (Zhang and Liu, 2011). Oral administration of kaempferol decreased fasting blood glucose and improved insulin resistance (Vinayagam and Xu, 2015). Other flavonoids with reported antidiabetic activity are naringin and naringenin, that are mainly found in various citrus fruits such as oranges and grapefruits (Alam et al., 2014). Several anti-diabetic mechanisms of naringenin were reported. In addition to its antioxidative potential and its inhibition of intestinal α-glucosidase it also exhibits insulin mimetic effect to decrease polyprotein B secretion (Priscilla et al., 2014; Vinayagam and Xu, 2015; Roy et al., 2016). Naringin effects expression of hepatic genes involved in gluconeogenesis and lipid metabolism and thus prevents the development of metabolic syndrome by activating AMPK (Pu et al., 2012). One of the most widely used and widely distributed flavonols in human nutrition with reported anti-diabetic and anti-inflammatory activity is quercetin (Vinayagam and Xu, 2015; Li et al., 2016). Several mechanisms of its action include inhibition of intestinal glucose absorption by inhibition of GLUT2 (Kwon et al., 2007), decrease of lipid peroxidation (Coskun et al., 2005), inhibition of α-amylase and α-glucosidase (Meng et al., 2016). With the rapid increase of diabetes incidence, a need for effective phytochemicals with anti-diabetic activity rises. Given the increasing evidence of anti-diabetic activity of flavonoids, it is plausible that consumption of flavonoid-rich foods can reduce the risk of diabetes and that flavonoids can be the potential drugs of choice in management of diabetes mellitus.

## Conclusion

Many over-the-counter dietary supplements that still have insufficient medical information and supporting scientific evidence are used in the treatment of patients with diabetes and related metabolic disorders. In majority of the herbal products and secondary metabolites used in treating diabetes, the mechanisms of action involve regulation of insulin signaling pathways, translocation of GLUT-4 receptor and/or activation the PPARγ as well as anti-inflammatory and immunomodulatory action. Although numerous *in vitro* and *in vivo* studies, and a number of clinical studies, have reported beneficial effects of various extracts and preparations from plants, the data remain conflicting. The knowledge in this field is still limited and further studies into identifying active ingredients of several botanicals and their extracts with reported antidiabetic activity, as well as unveiling their mechanism of action, are needed. Since plants and their extracts constitute numerous active ingredients with unknown effect, caution is needed when interpreting and generalizing antidiabetic properties of such preparations. Synergistic effect of different phyto-derived constituents must also be considered. We must also take into consideration that many phytochemicals taken orally undergo considerable loss of bioactivity. Several novel delivery systems, based on natural materials, are already being developed to counter this problem and increase bioavailability of phyto-derived antidiabetic compounds. More studies are warranted to validate their efficacy and safety, and large, well-designed, randomized, double-blind, placebo-controlled, clinical studies need to be carried out before their use can be recommended for treatment and/or prevention of diabetes. As diabetes incidence increases, a need for effective phytochemicals with anti-diabetic activity increases and growing evidence of anti-diabetic activity of several plants or their constituents provides a great pool of potential effective and safe drug for prevention and management of diabetes.

## Author Contributions

NPU contributed to idea, writing, editing, and financial support. AO performed writing.

## Conflict of Interest Statement

The authors declare that the research was conducted in the absence of any commercial or financial relationships that could be construed as a potential conflict of interest. The reviewer YW and handling Editor declared their shared affiliation, and the handling Editor states that the process met the standards of a fair and objective review.
